# Establishment of iPSC-Derived MSCs Expressing hsa-miR-4662a-5p for Enhanced Immune Modulation in Graft-Versus-Host Disease (GVHD)

**DOI:** 10.3390/ijms26020847

**Published:** 2025-01-20

**Authors:** Susie Lee, Eung-Won Kim, Hae-Ri Lee, Sun-Ung Lim, Chan Kwon Jung, Young-Ju Kang, Gyung-Ah Jung, Il-Hoan Oh

**Affiliations:** 1 Catholic High-Performance Cell Therapy Center & Department of Medical Life Science, College of Medicine, The Catholic University of Korea, Seocho-gu, Seoul 06591, Republic of Korea; 2 Department of Medical Sciences, College of Medicine, The Catholic University of Korea, Seocho-gu, Seoul 06591, Republic of Korea; 3 RegenInnopharm Inc., Seocho-gu, Seoul 06591, Republic of Korea; 4 Department of Pathology, Seoul St. Mary’s Hospital, College of Medicine, The Catholic University of Korea, Seocho-gu, Seoul 06591, Republic of Korea

**Keywords:** graft-versus-host disease, induced pluripotent stem-cell-derived mesenchymal stem cell, microRNA

## Abstract

The immune-modulatory effects of mesenchymal stromal cells (MSCs) are widely used to treat inflammatory disorders, with indoleamine 2,4-dioxygenase-1 (IDO-1) playing a pivotal role in suppressing stimulated T-cell proliferation. Taking that three-dimensional (3D) cultures enhance MSCs’ anti-inflammatory properties compared with two-dimensional (2D) cultures, the differentially expressed miRNAs were examined. Thus, we identified hsa-miR-4662a-5p (miR-4662a) as a key inducer of IDO-1 via its suppression of bridging integrator-1 (BIN-1), a negative regulator of the IDO-1 gene. The IDO-1-inducing potential of miR-4662a was conserved across primary MSCs from various donors and sources but exhibited variability. Notably, iPSC-derived MSCs (iMSCs) demonstrated superior IDO-1 induction and immune-modulatory efficacy compared with their donor-matched primary MSCs. Accordingly, iMSCs expressing miR-4662a (4662a/iMSC) exhibited stronger suppressive effects on T-cell proliferation and more potent suppressive effects on graft-versus-host disease (GVHD), improving survival rates and reducing tissue damage in the liver and gut. Our results point to the therapeutic potential of standardized, off-the-shelf 4662a/iMSC as a robust immune-modulating cell therapy for GVHD.

## 1. Introduction

Mesenchymal stromal cells (MSCs) are a non-hematopoietic cell population characterized by their multi-lineage differentiation potential and paracrine-supporting roles in tissue repair and regeneration [1,2,3]. To date, over 425 clinical trials have explored the therapeutic applications of cultured MSCs in tissue regeneration [4]. These therapeutic effects are largely attributed to the paracrine actions of MSCs, mediated by the secretion of growth factors and cytokines that promote endogenous stem-cell proliferation, angiogenesis, and survival signaling [5,6,7]. Another critical function of MSCs is their ability to modulate immune responses, primarily through their immune-suppressive and anti-inflammatory activities, which help limit inflammation in injured tissues [8,9]. Consequently, MSCs have been widely investigated for treating inflammatory diseases, such as graft-versus-host disease (GVHD) and Crohn’s disease, and for inducing immune tolerance in organ transplantation [10,11,12,13].

GVHD is an autoimmune condition triggered by donor T-cell responses against host tissues, including the liver, gut, and skin, following allogeneic hematopoietic stem-cell transplantation (HSCT) for malignant or non-malignant disorders [13]. Although corticosteroids remain the first-line treatment for GVHD, approximately 50% of patients are refractory to steroid therapy [14], making GVHD a significant cause of morbidity and mortality post-HSCT.

MSCs have demonstrated their ability to suppress immune responses in both preclinical and clinical studies of GVHD by mitigating the efferent phase of the disease [15,16,17]. These studies have shown promising results in immune suppression and therapeutic efficacy. However, MSC-based clinical trials have been hindered by inconsistent outcomes due to the heterogeneity of MSCs derived from different donors and tissue sources [18,19,20]. Functional variability in MSCs also arises during their in vitro culture [21,22,23], leading to inconsistent biological effects. Although MSCs have been shown to alleviate steroid-resistant GVHD in clinical trials [16], phase III studies using industrial MSC products have yielded suboptimal results, necessitating further investigation into MSC-mediated immune modulation [24,25,26].

The immune-modulating effects of MSCs influence both innate and adaptive immune responses. The MSCs are activated by inflammatory cytokines such as interferon-gamma (IFN-γ), tumor necrosis factor-alpha (TNF-α), or interleukin-1 (IL-1) and produce indoleamine 2,3 dioxygenase1 (IDO-1) to suppress the proliferation of T-cells [8,27,28]. Alternatively, MSCs secrete prostaglandin E2 (PGE2) to induce anti-inflammatory macrophages or secrete tumor necrosis factor-inducible gene 6 (TSG-6) that interacts with CD44 in macrophages to inhibit toll-like receptor 2 (TLR2)/nuclear factor kappa-light-chain enhancer of activated B cells (NF-κB) signaling to reduce proinflammatory cytokines. These mechanisms collectively inhibit both the early and efferent phases of inflammation [29].

Studies have shown that IFN-γ, a cytokine produced by activated T-lymphocytes, significantly enhances MSC motility and cytotoxicity [30,31]. The immune-suppressive activities of MSCs are modulated by factors within the inflammatory microenvironment and are particularly dependent on IFN-γ-mediated MSC activation, which upregulates IDO-1 [28,32]. This enzyme catalyzes the degradation of tryptophan to kynurenine, suppressing T-cell proliferation in the mid-G1 phase and inhibiting effector T-cell activity [33]. Therefore, IDO-1 has emerged as a critical potency marker for MSCs in treating inflammatory diseases [8].

Notably, accumulating studies have shown that MSCs cultured in three-dimensional (3D) spheroid cultures exhibit enhanced anti-inflammatory activities, with higher levels of cytokines controlling the immune response [34,35,36]. Moreover, MSCs under 3D cultures exhibit changes in their stemness and the depth of the epithelial–mesenchymal transition (EMT gradient), thereby exhibiting enhanced therapeutic potential [37]. Notably, these functional enhancements are closely associated with altered microRNA (miRNA) expression profiles [37], underscoring the regulatory role of miRNAs in MSC behavior [9,38].

In this study, we hypothesized that the superior anti-inflammatory effects of 3D MSCs are linked to their distinct miRNA expression profiles. We aimed to identify miRNAs that could induce IDO-1 in MSCs and thereby enhance their immune-modulatory capabilities. Through a comprehensive screening process, we identified hsa-miR-4662a-5p (miR-4662a) as a potent inducer of IDO-1 in MSCs. We further investigated the reproducibility of this phenomenon across various MSC sources and examined its potential to improve the immune-modulatory effects of induced pluripotent stem cell (iPSC)-derived MSCs (iMSCs). Our findings highlight the potential of iMSCs expressing miR-4662a (4662a/iMSC) as a standardized, off-the-shelf cell therapy for treating inflammatory diseases like GVHD.

## 2. Results

### 2.1. Identification of miRNAs Differentially Expressed in 3D Cultures Compared with 2D MSCs

**Figure 1:**
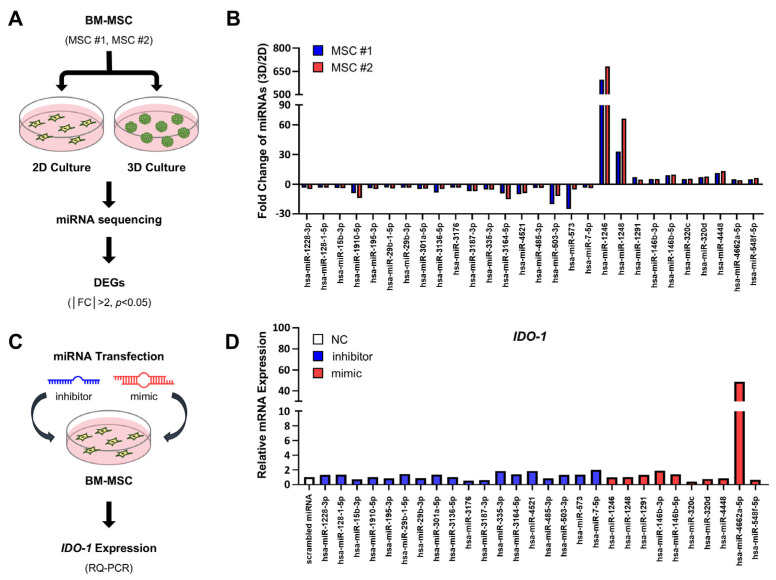
Identification of miR-4662a as an immune-modulating factor in MSCs. (**A**) Schematic diagram of the process for identifying miRNAs differentially expressed in 2D and 3D MSCs. Two independent donor-derived MSCs, #1 and #2, were cultured in either plastic adherent (2D) or spheroid (3D) conditions for 48 h. RNA sequencing was conducted to identify miRNAs with significant differential expressions (|fold change| > 2; *p* < 0.05). (**B**) Fold changes in miRNAs differentially expressed between 2D and 3D MSCs from two independent donors are shown (n = 1). (**C**) Schematic diagram of the process for analyzing the immune-modulating effects of the differentially expressed miRNAs. The MSCs were transfected with agomiR (mimic) for upregulated miRNAs or with antagomiR (inhibitor) for downregulated miRNAs in (**B**), then analyzed for changes in the expression levels of *IDO-1* by using RQ-PCR. (**D**) Changes in the expression levels of *IDO-1* from 28 candidate mimics/inhibitors in (**B**) after normalization to *HPRT*. Shown are the fold changes in expression levels for each mimic/inhibitor compared with the scrambled negative controls.

To identify miRNAs potentially contributing to the enhanced anti-inflammatory properties of three-dimensional (3D) MSC cultures compared with two-dimensional (2D) cultures, we analyzed miRNA expression profiles from the bone-marrow-derived MSCs (BM-MSCs) of two independent donors. Each donor’s MSCs were cultured in 3D spheroids and 2D adherent conditions, and their miRNA profiles were compared (Figure 1A). A total of 28 miRNAs were differentially expressed between the two conditions (|fold change| > 2; *p* < 0.05), with 18 downregulated and 10 upregulated miRNAs in 3D MSCs. Notably, similar expression patterns were observed across both donors (Figure 1B).

To explore the effects of these differentially expressed miRNAs on immune modulation, we determined their effects on *IDO-1*, taking that it serves as a major immune modulatory factor and that inducibility of IDO-1 has been considered as a potency marker for MSCs for inflammatory disease [8,39]. Inhibitors (antisense oligonucleotides) targeting the 18 downregulated miRNAs and mimics (miRNA agomiR) for the 10 upregulated miRNAs were transfected with 2D MSCs to replicate the 3D culture expression profile (Figure 1C). miR-4662a was identified as the only miRNA capable of markedly upregulating *IDO-1* expression among the tested candidates, suggesting its crucial role in modulating MSC immune properties (Figure 1D).

### 2.2. Donor Variations in IDO-1-Inducing Effects of miR-4662a

**Figure 2:**
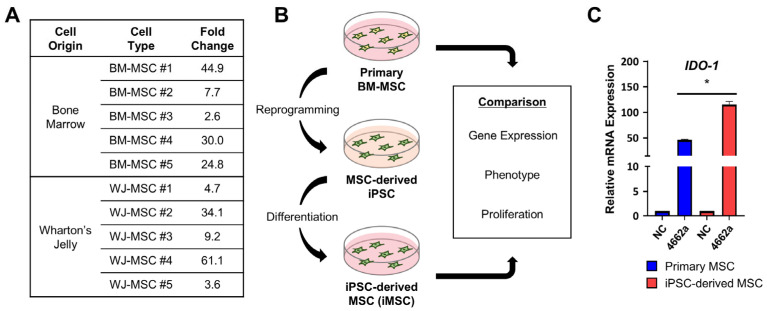
Comparisons of *IDO-1* induction in various sources of MSCs by miR-4662a. (**A**) The fold induction of *IDO-1* expression was compared among the bone marrow or Wharton-jelly-derived MSCs obtained from multiple donors. Fold changes in *IDO-1* in primary MSCs from each indicated donor were determined using RQ-PCR, normalized to *HPRT*, and ascertained in comparison to scrambled control (n = 2 for each donor cell). (**B**) Schematics for the generation of donor-matched iPSC-derived MSCs (iMSC) and studies comparing the primary and donor-matched iMSCs. (**C**) Comparison of the *IDO-1*-inducing potential of MSCs from primary BM-MSCs and donor-matched iMSCs. *IDO-1* expression in primary MSCs or iMSCs after transfection with miR-4662a was assessed using RQ-PCR. Data were normalized to *HPRT* and are presented as fold changes relative to scrambled miRNA controls (n = 3 for each group; * *p* < 0.05).

After confirming miR-4662a’s ability to enhance IDO-1 expression in primary BM-MSCs, we evaluated its effects across multiple donors and MSC sources, taking the high variations in MSCs with respect to the donors or differences in the ontological stage [40,41]. BM-MSCs from five donors and Wharton’s-jelly-derived MSCs from five additional donors were transfected with miR-4662a. All samples exhibited increased *IDO-1* expression, albeit to varying extents (ranging from 2.6-fold to 44.9-fold in BM-MSCs and a similar variability in Wharton’s-jelly-derived MSCs) (Figure 2A). This variability indicated that donor-variable factors primarily influenced miR-4662a’s efficacy rather than the ontological source of MSCs.

Therefore, to overcome donor-dependent variability, we explored the use of induced pluripotent stem-cell-derived MSCs (iMSCs). Induced pluripotent stem cells (iPSCs) have been shown to be an alternative source of MSCs, with higher ex vivo expandability and a standard characterization from established cell lines [37,42,43]. Accordingly, donor-matched primary BM-MSCs and their iMSCs were generated (see Figure 2B for the schematic), and these iMSCs exhibited a canonical phenotype of primary MSCs (Figure S1). Each type of MSC was then compared for their *IDO-1* induction folds by miR-4662a transfection. As shown in Figure 2C, *IDO-1* induction was significantly greater in iMSCs (115.5-fold) compared with their primary MSC counterparts (46.7-fold). This finding highlighted iMSCs as a promising platform for consistent, high-level *IDO-1* induction via miR-4662a.

### 2.3. Mechanism of IDO-1 Induction by miR-4662a

**Figure 3:**
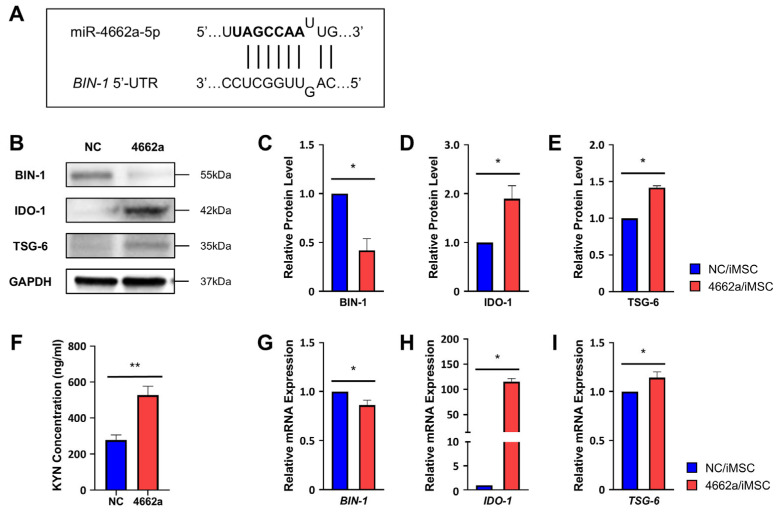
*BIN-1* serves as a target of miR-4662a for immunomodulatory effects. (**A**) Alignment of the miR-4662a sequence with the *BIN-1* 5′-UTR region. The matching miRNA seed sequence (marked in bold) with the 5′-UTR region of *BIN-1* is shown. (**B**–**E**) Protein levels of BIN-1, IDO-1, and TSG-6 in iMSCs transfected with a scrambled miRNA control (NC/iMSC) or miR-4662a mimic (4662a/iMSC) were analyzed using Western blotting. Shown are the representative plots for the Western blot (**B**) and their quantification for expression levels normalized to GAPDH, presented as fold changes relative to scrambled miRNA controls (**C**–**E**) (n = 3 for each group; * *p* < 0.05). The samples were derived from the same experiment and the gels/blots were processed in parallel. Full-length images are presented in Figure S2. (**F**) Kynurenine (KYN) concentration in the culture medium of iMSCs transfected with miRNA (NC or miR-4662a). The concentration of KYN, the metabolite of *IDO-1*, was analyzed using an ELISA (n = 5; ** *p* < 0.01). (**G**–**I**) Quantitative analysis for expression levels of the transcripts for *BIN-1*, *IDO-1*, and *TSG-6* was analyzed using RQ-PCR for NC/iMSC or 4662a/iMSC (n = 3 or 4; * *p* < 0.05). Data were normalized to *HPRT* and are shown as fold changes in transcript levels relative to scrambled controls.

To elucidate the mechanism by which miR-4662a induces IDO-1, we hypothesized that it targets a negative regulator of the *IDO-1* gene to de-repress it. A bioinformatic analysis identified bridging integrator-1 (*BIN-1*) as a potential miR-4662a target based on sequence complementarity within its 5′ untranslated region (UTR) (Figure 3A). BIN-1, previously characterized as a tumor-suppressor gene [44], has been shown to act as a transcriptional repressor of the IDO-1 gene through pathways involving signal transducers as well as activator of transcription 1 (STAT1) and NF-κB [27,45]. Notably, BIN-1 has also been identified as an immune-potentiating factor within the tumor microenvironment [46,47]. Based on these findings, we anticipated that *BIN-1* would play a critical role in controlling IDO-1 expression in iMSCs and thus serve as a key target for miR-4662a-mediated modulation.

Consistent with this prediction, miR-4662a transfection in iMSCs (4662a/iMSC) suppressed BIN-1 expression at both transcript and protein levels (Figure 3B,C,G) compared with the scrambled miRNA-transfected group (NC/iMSC). Concurrently, the IDO-1 protein, its gene expression (Figure 3B,D,H), and its metabolite, kynurenine (KYN) (Figure 3F) were significantly elevated, indicating that miR-4662a’s *IDO-1* induction occurred via *BIN-1* suppression. Additionally, we examined if miR-4662a influenced other immune-modulatory factors. The expression of TSG-6, an anti-inflammatory mediator, was markedly upregulated in 4662a/iMSC at both mRNA and protein levels (Figure 3B,E,I), consistent with a previous study showing that the kynurenic acid produced from *IDO-1* can enhance the production of TSG-6 [48].

**Figure 4:**
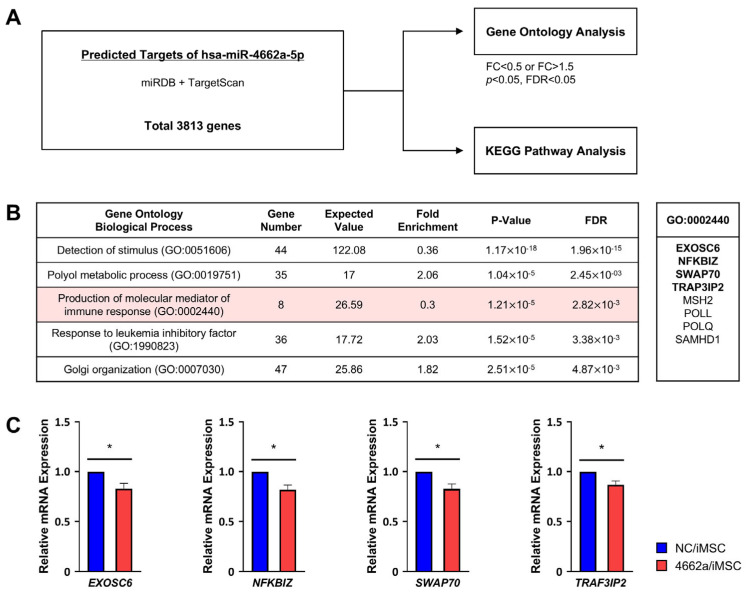
Gene ontology analysis of miR-4662a for additional immunomodulatory effects. (**A**) Schematic outline of bioinformatic analysis of immune-modulating effects of miR-4662a. The analysis of gene ontology (GO) and KEGG pathways used miRDB and TargetScan databases, with PantherDB and KEGG Mapper used for pathway visualization. (**B**) Left: GO analysis for biological process. The top five GO terms with the significance of FDR are shown. The GO (GO:0002440) for factors related to the immune response is highlighted in red. The full GO analysis results are presented in Table S3 (FC < 0.5 or >1.5; *p* < 0.05; FDR < 0.05). Right: The genes predicted in GO for immune-modulating effects (GO:0002440) are listed, with experimentally analyzed genes marked in bold. (**C**) Expression levels of immune-modulating genes (GO:0002440) were analyzed using RQ-PCR in iMSCs transfected with miR-4662a (n = 3 or 4; * *p* < 0.05). Data were normalized to *HPRT* and are presented as fold changes relative to scrambled controls.

In addition, through the bioinformatics approach, we analyzed the gene ontology (GO) of the target genes identified in the databases miRDB and TargetScan (Figure 4A). It showed the significant (*p* < 0.05) enrichment of gene clusters participating in immune modulation (Figure 4B), with the downregulation of individual genes in the GO for immune-modulating genes such as *EXOSC6* [49], *SWAP70* [50], *TRAF3IP2* [51], or *NFKBIZ* [52,53]. As a result, the GO analysis further suggested that miR-4662a targets multiple immune-regulatory pathways, supporting its additional role in modulating broader immune responses.

Similarly, the KEGG analysis of the gene sets for miR-4662a target genes for immune regulation suggested multiple sites of action in the complex immune-regulatory network in the antigen presentation, including cluster of differentiation 28 (*CD28*), MHC-related genes (*HLA-A* and *HLA-E*), or *IL-6* (Figure S3). Moreover, 4662a/iMSC expressed higher levels of cytokines known to mediate the anti-inflammatory function of MSCs, such as cyclooxygenase-2 (*COX2*), hepatocyte growth factor (*HGF*), transforming growth factor-beta 1 (*TGF-β1*), or interleukin-10 (*IL-10*) [29,54] (Figure S4).

Together, these results indicated that the immune-modulating effect of miR-4662a should contribute to multiple immune mechanisms in addition to their IDO-1/TSG-6 axis.

### 2.4. Anti-Proliferative Effects on T-Cells

**Figure 5:**
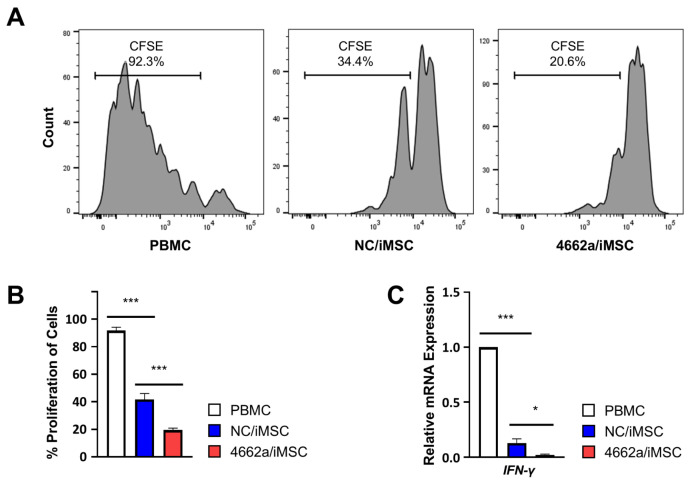
Immune-modulating effects of miR-4662a on T-cells. (**A**,**B**) Inhibition of T-cell proliferation by 4662a/iMSC. Peripheral blood mononuclear cells (PBMCs) from normal donors were stained with CFSE and incubated with an absence of iMSCs (PBMC) or co-cultured with NC/iMSC or 4662a/iMSC for 6 days. The decrease in the CFSE intensity by mitotic divisions of T-cells was analyzed using flow cytometry after gating for T-cells (CD3) (CFSE cells; n = 4; *** *p* < 0.001). (**C**) *IFN-γ* expression of T-cells after 6 days of culture was measured using the culture above and RQ-PCR (n = 3; * *p* < 0.05; *** *p* < 0.001). Gene expression levels were normalized to HPRT and are presented as fold changes relative to the PBMC group.

Given miR-4662a’s impact on immune-gene expression, we evaluated its functional effects on T-cell proliferation. Peripheral blood mononuclear cells (PBMCs) were co-cultured with NC/iMSC or 4662a/iMSC under CD3-stimulated conditions. The 4662a/iMSC sample significantly inhibited T-cell proliferation, as evidenced by a higher retention of CFSE-labeled T-cells compared with the co-culture with NC/iMSC or the culture with an absence of iMSCs (PBMC) (Figure 5A,B and Figure S5). Similarly, *IFN-γ* production in activated T-cells was significantly reduced in the presence of 4662a/iMSC compared with the PBMC or NC/iMSC groups (Figure 5C).

### 2.5. Effects of miR-4662a on the Suppression of GVHD

**Figure 6:**
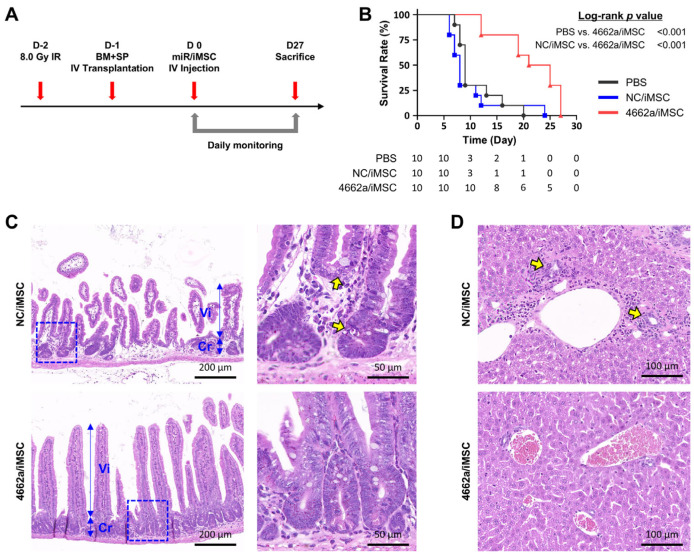
Suppression of GVHD by 4662a/iMSC in GVHD-model mice. (**A**) Schematic diagram of the experiment for production of GVHD mouse model analysis of therapeutic effects. Allogenic bone marrow (BM) and splenocytes (SPs) from C57BL/6 mice were transplanted into BALB/c recipients 24 h after irradiation (8.0 Gy IR). Then, 24 h after transplantation, recipient mice were administered iMSCs transfected with a scrambled control or miR-4662a. Daily monitoring was conducted throughout the experiment. (**B**) Survival rates of the GVHD mice treated with 4662a/iMSC or NC/iMSC (n = 10 for each group). The log-rank *p*-value of the survival rate is presented on the right side of the graph. The time-dependent number of risks table is shown at the bottom of the survival curve. (**C**) Histological analysis of intestines in GVHD mice treated with each group of iMSCs. Shown are the hematoxylin and eosin (H&E)-stained intestinal sections of GVHD mice treated with NC/iMSC (upper panels) or 4662a/iMSC (lower panels). The double-headed arrows within the left panels indicate the individual lengths of the villus (Vi) and crypt (Cr). The right panels are 4X magnification images of the boxed inlet region in the left panels. Apoptotic cells and karyorrhectic debris are indicated by yellow arrows (scale bars: left, 200 μm; right, 50 μm). (**D**) Histology of the liver from H&E staining of GVHD mice. Bile-duct damage and portal inflammation are indicated by yellow arrows (scale bar: 100 μm).

Based on the observed suppressive effects of 4662a/iMSC on T-cells, we next evaluated its impact on GVHD murine models. GVHD was induced in mice by the transplantation of allogeneic bone marrow (BM) cells and splenocytes (SPs), followed by an injection of 4662a/iMSC into the transplanted mice (see Figure 6A for the schematics). Recipient mice that did not receive any iMSC injections (PBS) showed a progressive decline in survival beginning 8 days post-transplantation. Similarly, mice treated with NC/iMSC exhibited a gradual reduction in survival between 8 and 24 days after transplantation (Figure 6B). In contrast, mice injected with 4662a/iMSC demonstrated 100% survival up to 12 days post-transplantation and significantly improved survival rates compared with the mice treated with PBS or NC/iMSC.

To further investigate the effects of 4662a/iMSC on GVHD activity in target tissues, we analyzed the histology of the gastrointestinal (GI) tract and liver from recipient mice treated with either NC/iMSC or 4662a/iMSC. In mice injected with NC/iMSC, the small intestine displayed severe crypt architectural distortion and crypt loss, along with notable villous atrophy, resulting in a villus-to-crypt ratio of 2.5. Histological examinations also revealed apoptotic cells and karyorrhectic debris. In contrast, small-intestine samples from mice treated with 4662a/iMSC showed well-preserved villus and crypt structures, with a villus-to-crypt ratio of 5.5. High-magnification images highlighted intact Paneth cells and glandular cells within the crypts (Figure 6C). Similarly, liver tissue from mice injected with NC/iMSC displayed damage to bile ducts, along with portal inflammation and fibrosis. However, liver sections from mice treated with 4662a/iMSC showed well-maintained bile ducts and portal veins, with no signs of portal inflammation (Figure 6D).

Collectively, these findings demonstrate that 4662a/iMSC significantly suppressed GVHD activity and provided enhanced therapeutic effects compared with control treatments.

## 3. Discussion

The anti-inflammatory effects of mesenchymal stromal cells (MSCs) have been widely applied to treat various diseases, including graft-versus-host disease (GVHD) and Crohn’s disease, and to induce tolerance in organ transplantation [10,11,12,13]. Among these, GVHD has been the most actively explored, given its role as a major cause of morbidity and mortality following hematopoietic stem-cell transplantation (HSCT). In both preclinical and clinical models, MSCs have shown immune-suppressive effects that alleviate the severity of GVHD [15,16,17]. However, despite these promising outcomes, phase III clinical trials using industrially manufactured MSC products have produced inconsistent results, emphasizing the need for improved strategies to enhance MSC-mediated immune modulation [24,25,26].

In this study, we aimed to enhance the therapeutic potential of MSC-based therapies for inflammatory disorders using GVHD as a model. One approach was to exploit the anti-inflammatory benefits of miRNAs, inspired by findings that three-dimensional (3D)-cultured MSCs exhibit higher anti-inflammatory effects [34,35,36]. We identified miR-4662a as a key regulator that upregulates *IDO-1*, a critical anti-inflammatory factor in MSCs known to suppress T-cell proliferation and serve as a potency marker for inflammatory disease therapy [8].

Another strategy was to address the variability in MSC function by utilizing induced pluripotent stem-cell-derived MSCs (iMSCs). Although miR-4662a has consistently enhanced IDO-1 production in primary MSCs derived from multiple donors, the magnitude of this effect significantly varies due to donor-specific differences. Moreover, primary MSCs exhibit a decline in proliferative activity with an increase in age and underlying disorders [55,56] as well as replicative senescence during the culture itself and associated gene expression changes [57]. iMSCs, on the other hand, offer a more reproducible platform, with higher expandability and uniformity [37,42,43,58]. We found that miR-4662a’s *IDO-1*-inducing effects were amplified in iMSCs compared with donor-matched primary MSCs, demonstrating the feasibility of developing standardized iMSC-based therapeutic cell lines for inflammatory diseases.

Accordingly, when examined for therapeutic effects, 4662a/iMSC exerted a stronger suppression of T-cell proliferation and a higher therapeutic potential for GVHD, exhibiting higher survival in the recipient animals and a stronger inhibition of pathological changes in their target organs. Thus, iMSC-based cell lines expressing miR-4662a could be an approach for more potent therapeutic effects and more reproducible outcomes in cell therapy for GVHD than previous approaches based on unmodified primary MSCs, opening the possibility for the off-the-shelf use of the cell therapy for GVHD. However, further verification of the efficacy in different types of GVHD, including acute and chronic models of GVHD, is warranted.

**Figure 7:**
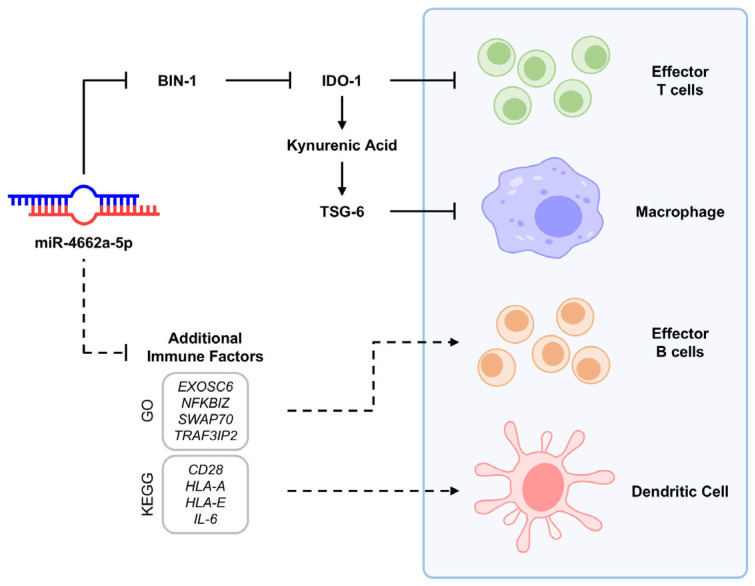
Schematic representation of the multiple immune-modulating effects of miR-4662a to suppress GVHD. miR-4662a targets BIN-1, the negative regulator of IDO-1, to cause the inhibition of T-cell proliferation. With the induction of IDO-1, its metabolite kynurenic acid is increased, causing upregulation of TSG-6, thereby inhibiting inflammation by macrophages. Bioinformatic analysis of miR-4662a target gene by GO analysis and KEGG pathway suggested additional immune-modulating effects with decreased expression of the genes for B-cell response (*EXOSC6*, *NFKB1Z*, *SWAP70*, and *TRAF3IP2*) and antigen presentation (*CD28*, *HLA-A*, *HLA-E*, and *IL-6*).

Of note, the mechanism underlying miR-4662a-dependent immune suppression was influenced by multiple regulatory mechanisms (Figure 7). Firstly, we observed significant suppression of *BIN-1*, the negative regulator of *IDO-1* [27,45], thereby inducing *IDO-1* in MSCs. Additionally, kynurenic acid, a metabolite of IDO-1, was shown to enhance TSG-6 expression [34], another critical anti-inflammatory mediator for macrophages [48]. Beyond the IDO-1/TSG-6 axis, gene ontology (Figure 4) and KEGG analyses (Figure S3) suggested that miR-4662a influenced multiple immune-regulatory pathways, including those involved in cytokine signaling and antigen presentation. This broad spectrum of activity underscores the potential of miR-4662a to modulate immune responses through diverse mechanisms.

Notably, although NC/iMSC and 4662a/iMSC both demonstrated significant suppressive effects on T-cell proliferation in the in vitro assay, only 4662a/iMSC exhibited significant suppressive effects on GVHD in the in vivo study model, revealing discrepancies between the two models. The underlying causes of these discrepancies remain unclear. However, controversies exist regarding the differences in immune suppression by MSCs, depending on the disease model [59,60]. Furthermore, the immunosuppressive function of MSCs is activated (“licensed”) by multiple cytokine stimuli such as IFN-γ or TNF-α [8,30,61,62]. Consequently, MSCs under in vivo conditions are exposed to microenvironments different from those under in vitro conditions, including cytokines and interactions with other cell types.

In our investigation, we examined the potential effectors that could differentially influence MSCs in vivo compared with in vitro, including the upregulation of multiple cytokine types. However, the functional significance of these additional immune effectors during the in vivo GVHD process necessitates further studies for a more precise elucidation of the role of each effector element. Further investigation is warranted to explore the complete spectrums of the immune-modulating effects of miR-4662a during in vivo immune responses.

Although our findings demonstrate the promising therapeutic potential of 4662a/iMSC, further studies are warranted to explore its efficacy in different GVHD models, including acute and chronic forms of the disease. Additionally, understanding the long-term safety and stability of 4662a/iMSC in clinical settings is critical for its translation into widespread clinical use.

In conclusion, 4662a/iMSC represents a significant advancement in immune-modulating cell therapies. Their ability to provide consistent, potent, and reproducible therapeutic effects offers a compelling solution to the challenges associated with traditional MSC-based treatments. This study paves the way for the development of standardized, off-the-shelf cell therapies for GVHD and, potentially, other inflammatory diseases.

## 4. Materials and Methods

### 4.1. Human BM-MSC Isolation and Culture

Human bone marrow (BM) MSCs derived from healthy donors were obtained from healthy donors with informed consent, following approval by the Institutional Review Board of the Catholic University of Korea (MC19SNSI0059; 9 May 2019). BM-MSCs were cultured in low-glucose Dulbecco’s modified Eagle’s medium (DMEM) (Hyclone Laboratories Inc., Schenectady, NY, USA) supplemented with 15% fetal bovine serum (FBS) (Hyclone), 100 U/mL penicillin, 100 mg/mL streptomycin, and L-glutamine in a humidified atmosphere of 5% CO_2_ at 37 °C.

### 4.2. Human iPSC Generation and Culture

For iPSC reprogramming, BM-MSCs were electroporated with Episomal iPSC Reprogramming Vectors (Invitrogen, Carlsbad, CA, USA) using the Neon Transfection System (Invitrogen) under optimized conditions (pulse voltage: 1650 V) following the manufacturer’s instructions. Post-electroporation, the cells were seeded into two sets of 35 mm dishes coated with Matrigel (Corning Inc., Corning, NY, USA) and cultured with mTeSR1 medium (Stem Cell Technologies, Vancouver, BC, Canada). To enhance reprogramming efficiency, 1 mM nicotinamide was added during this process. After 14–21 days, ESC-like iPSC colonies were selected, passaged, and expanded for subsequent characterization.

### 4.3. iMSC Generation and Culture

For differentiation into MSCs, iPSC colonies were grown to confluence in Matrigel-coated dishes in mTeSR2 medium (Stem Cell Technologies) and switched to MSC differentiation media containing DMEM with 5 ng/mL basic fibroblast growth factor (bFGF) (Peprotech, Rocky Hill, NJ, USA), 0.1 mM non-essential amino acids (Gibco, Grand Island, NY, USA), 1% Glutamax (Gibco), 1% antibiotics (Gibco), and 10 µM SB431542 (Sigma-Aldrich, Carlsbad, CA, USA) in DMSO (Sigma-Aldrich). Media changes were performed every 2 days for 14 days, and iMSCs were maintained on 0.1% gelatin-coated tissue culture dishes. Cells were passaged to a single-cell suspension using Trypsin/EDTA (Gibco). iPSC-derived MSCs were cultured in DMEM with 15% FBS, L-glutamine, 100 U/mL penicillin, and 100 mg/mL streptomycin into 0.1% gelatin (Sigma-Aldrich)-coated tissue culture dishes for maintenance. Every step of isolating and generating human iMSCs was enforced as previously described [37].

### 4.4. Three-Dimensional Spheroid Generation

To generate 3D MSC spheroids, soft lithography techniques were used to create Polydimethylsiloxane (PDMS)-based concave microchips [63]. MSCs were seeded into microchips pre-coated with 3% (*w*/*v*) bovine serum albumin (BSA) (Sigma-Aldrich) to minimize cell attachment. After 24 h, the spheroids were transferred to low-adherence dishes (Corning) for another 24 h of culture suspension.

### 4.5. Flow Cytometry

Flow cytometry was performed on reprogrammed iMSCs to confirm the expression of canonical MSC markers. Cells were stained with the following antibodies: anti-human CD45-BV421, CD73-PE, CD90-FITC, and CD105-APC (BD Pharmingen, San Diego, CA, USA). They were then incubated on ice for 30 min before analysis using a BD LSR II flow cytometer (BD Biosciences, San Jose, CA, USA).

### 4.6. CFU-F Assay

MSCs and iMSCs were plated as 1 × 10^3^ cells into 100 mm tissue culture dishes and cultured in DMEM containing 15% FBS, 100 U/mL penicillin, 100 mg/mL streptomycin, and L-glutamine at 37 °C and 5% CO_2_. Colonies were fixed in methanol and stained with crystal violet (Sigma-Aldrich) after 14 days.

### 4.7. miRNA Sequencing

For miRNA sequencing, RNA was extracted from 2D and 3D MSC cultures, denatured, and resolved on a 15% polyacrylamide gel. Small RNA fragments (18–26 nucleotides) were excised and eluted overnight in 0.5 M NaCl and then precipitated using ethanol. A small RNA library was prepared using the TruSeq Small RNA Sample Prep Kit v2 (Illumina, San Diego, CA, USA) with 1 μg RNA. For base-calling and the generation of raw, de-multiplexed sequencing data in FASTQ format, Illumina HCS version 1.4.8 (Illumina, San Diego, CA, USA), RTA version 1.12.4.2 (Illumina), and CASAVA version 1.8.2 (Illumina) software were used.

### 4.8. miRNA Transfection

iMSCs were seeded at 5 × 10^5^ cells/well in 6-well plates and transfected with 60 nM AccuTarget™ miRNA mimic (BIONEER, Daejeon, Republic of Korea) or a scrambled miRNA control using Oligofectamine™ reagent (Invitrogen). The miR-4662a mimic sequences were 5′: UUAGCCAAUUGUCCAUCUUUAG and 3′: CUAAAGAUGGACAAUUGGCUAA. The full list of the miRNA sequences used for the experiment is shown in Table S1. Scrambled miRNA was used as the negative control. Post-transfection, cells were cultured in DMEM/low glucose supplemented with 10% FBS, L-glutamine, 100 U/mL penicillin, and 100 mg/mL streptomycin for 3 days at 37 °C in 5% CO_2_. RNA, protein, and the culture medium were harvested for further analysis.

### 4.9. Western Blotting Assay

iMSCs were lysed with a Laemmli buffer (GenDEPOT, Katy, TX, USA), and protein concentrations were determined using a BCA protein assay kit (Thermo Fisher Scientific, North Chicago, IL, USA). Equal amounts of protein (20 μg/lane) were separated by electrophoresis and transferred onto a Trans-Blot turbo transfer pack (Bio-Rad, Hercules, CA, USA). The membranes were blocked with 7.5% Difco™ skim milk (BD, Detroit, MI, USA) and probed overnight at 4 °C with primary antibodies against GAPDH (Santa Cruz Biotechnology, Santa Cruz, CA, USA), BIN-1 (Invitrogen), IDO-1 (Santa Cruz Biotechnology), and TSG-6 (ABclonal, Woburn, MA, USA) dissolved in 1% BSA (Bovogen, Melbourne, Australia) in 1X PBS-T. The membranes were treated with a secondary anti-mouse IgG (Cell Signaling, Beverly, MA, USA) or anti-rabbit IgG (Cell Signaling) HRP-linked antibody dissolved in 7.5% Difco™ skim milk (BD). Protein bands were visualized using the SuperSignal™ West Femto Maximum Sensitivity Substrate (Thermo Fisher Scientific), and protein expression was quantified using a PXi4 image analyzer system (Syngene, Cambridge, UK). ImageJ version 1.54d (US National Institutes of Health, Bethesda, MD, USA) was used to quantify the expression levels of proteins.

### 4.10. Real-Time Quantitative Reverse Transcriptase Polymerase Chain Reaction (RQ-PCR)

Total RNA from MSCs and iMSCs was extracted using TRIzol™ reagent (Invitrogen) and was reverse-transcribed using ReverTraAce™ qPCR RT Master Mix (TOYOBO, Osaka, Japan). Quantitative PCR was performed using the QuantStudio 3 Real-Time PCR System (Applied Biosystems, Foster City, CA, USA) with the SYBR Green PCR Master Mix (Thermo Fisher Scientific). The RT-qPCR primer sequences used for the experiment are shown in Table S2. Gene expression was normalized to the HPRT expression, and the relative expression was calculated using the ΔΔCt method.

### 4.11. Enzyme-Linked Immunosorbent Assay (ELISA)

Kynurenine (KYN) levels in the culture medium of iMSCs were measured using a KYN ELISA kit (ImmuSmol, Bordeaux, France), following the manufacturer’s instructions. All tests were performed in duplicate. Absorbance was detected at 450 nm using a PowerWave XS microplate reader (BIOTEK, Winooski, VT, USA), and KYN concentrations were determined based on a standard curve.

### 4.12. Gene Ontology and KEGG Pathway

Two miRNA target prediction databases, miRDB [64] and TargetScan [65] were used to analyze the targets of miR-4662a. A total of 3813 genes were shown to be the target of miR-4662a (486 genes from miRDB and 3775 genes from TargetScan). Using the predicted 3813 target genes, gene ontology (GO) and KEGG pathway analyses were conducted. For the GO analysis, PantherDB (https://www.pantherdb.org/ (accessed on 16 October 2024)) [66] was used. The biological process (BP) was analyzed using a standard of fold change <0.5 or a fold change >1.5, a *p* value < 0.05, and an FDR < 0.05. For the analysis of the KEGG pathway, the gene symbols were transferred to ENTREZ gene ID via DAVID Bioinformatics (https://davidbioinformatics.nih.gov/tools.jsp (accessed on 17 October 2024)) [67]. The ENTREZ gene IDs were analyzed for the KEGG pathway using KEGG mapper (https://www.kegg.jp/kegg/mapper/color.html (accessed on 17 October 2024)) [68,69,70].

### 4.13. T-Cell Proliferation Assay

PBMCs collected from a single donor were purchased from CTL (ImmunoSpot, Cleveland, OH, USA), and were seeded in 24-well plates at a density of 1 × 10^5^ cells/well. iMSCs were seeded at 5 × 10^4^ cells/well in 6-well plates and were transfected with either the miR-4662a mimic or a scrambled miRNA control the day before PBMC co-culture. The lymphocytes in the PBMCs were stimulated by treating 50 ng/mL recombinant human IL-2 (Peprotech) and 10 μL/mL T-cell TransAct™ (Miltenyi Biotec, Bergisch-Gladbach, Germany). iMSCs and stimulated PBMCs (1:10 ratio) were co-cultured for 6 days, and the proliferation of lymphocytes as well as *IFN-γ* secretion were measured. The cell proliferation analysis was performed using carboxyfluorescein succinimidyl ester (CFSE) (Invitrogen) dye, following the manufacturer’s instructions. The acquisition was performed using a BD FACSLyric Cytometer (BD Biosciences) and analyzed using FlowJo v10 software (FlowJo, Ashland, OR, USA).

### 4.14. Experimental GVHD Model

All animal experiments were conducted in compliance with the Laboratory Animals Welfare Act and approved by the IACUC of the Catholic University of Korea (approval number: CUMS-2022-0320-01). Six-week-old male C57BL/6 and BALB/c mice were purchased from Orient Bio Inc. (Seongnam, Republic of Korea) and housed under SPF conditions. Although BALB/c mice were used as recipients, C57BL/6 mice were chosen for grafts of bone marrow (BM) and splenocyte (SP) cells to be transplanted. After 7 days of quarantine, BALB/c mice were randomly divided into three groups (n = 10/group). Then, 8.0 Gy-irradiated BALB/c mice were intravenously injected with 5 × 10^6^/100 μL BM cells and 5 × 10^6^/100 μL SP cells (total: 200 μL/mouse) from C57BL/6 donors. Two days post-irradiation, 5 × 10^5^ iMSCs transfected with the miR-4662a mimic or scrambled miRNA were administered. The survival rates were recorded throughout the experiment.

### 4.15. Histopathological Analysis

Liver and intestine tissues from GVHD-model mice were fixed in 4% paraformaldehyde (PFA) (Biosesang, Seongnam, Republic of Korea) and then paraffin-embedded. Hematoxylin and eosin (H&E) staining was performed on 4 µm thick sections, and slides were examined using a Panoramic SCAN II slide scanner (3DHISTECH Ltd., Budapest, Hungary). The status of liver and intestinal tissues was assessed using Slide Converter (3DHISTECH Ltd.).

### 4.16. Statistical Analysis

Statistical significance was determined using a one-way ANOVA with Dunnett or Tukey multiple comparisons tests for three or more groups, and unpaired two-tailed *t*-tests for comparisons between the two groups. Statistical significance was defined as *p* < 0.05. The survival curve was analyzed using the log-rank *p*-value statistics. The statistical analysis was accomplished using Prism 8 (GraphPad Software, San Diego, CA, USA).

## Data Availability

Data are contained within the article and Supplementary Materials. The data presented in this study are available on request from the corresponding author.

## Supplementary Materials

The following supporting information can be downloaded at: https://www.mdpi.com/article/10.3390/ijms26020847/s1.
