# Supplementary material for: Establishment of iPSC-Derived MSCs Expressing hsa-miR-4662a-5p for Enhanced Immune Modulation in Graft-Versus-Host Disease (GVHD)
Source: Int J Mol Sci. 2025 Jan 20;26(2):847. doi: 10.3390/ijms26020847 (PMC11766046; doi:10.3390/ijms26020847)
Supplement: Supplementary file 1 [file ijms-26-00847-s001.zip › ijms-3423274-supplementary.pdf]

Supplementary Materials

Figure S1. Reprogramming BM-MSCs into iPSCs and generating iPSC-derived MSCs.

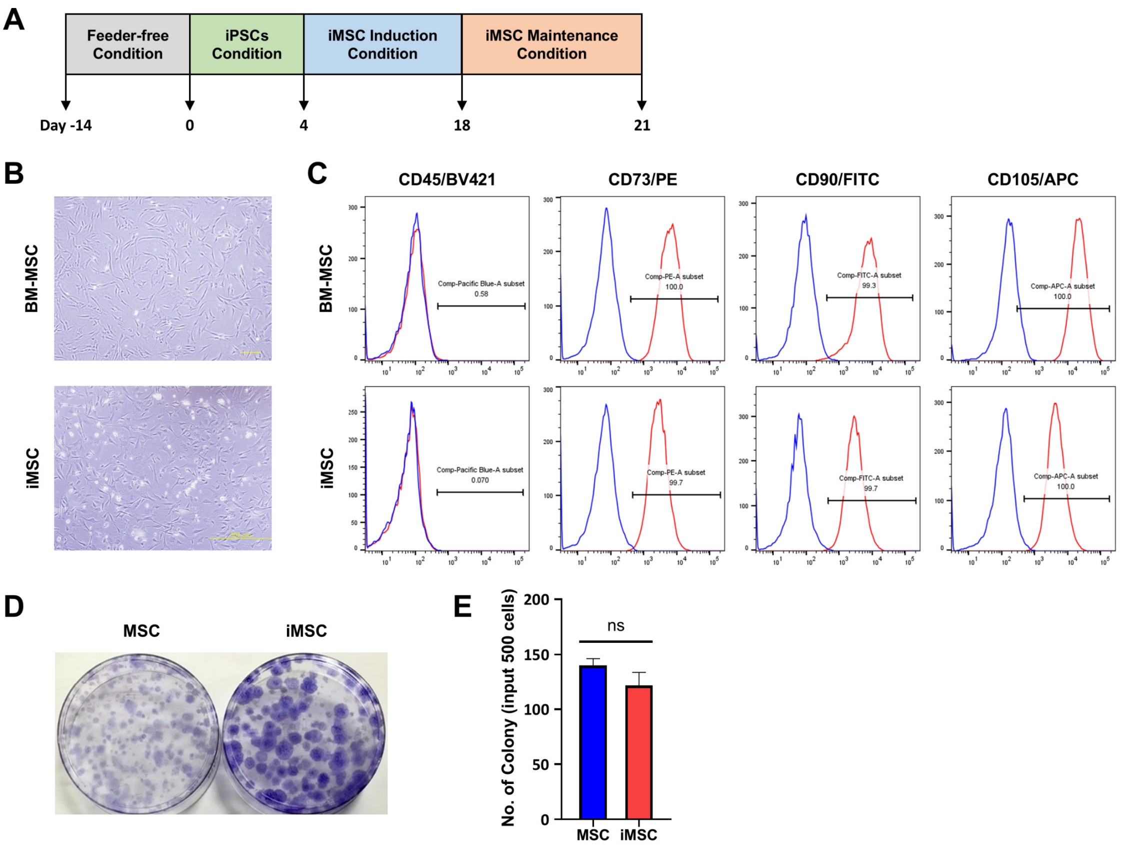

(A) Schematic representation of the process for establishing iMSC lines derived from donor-matched iPSCs. **(B)** Bright-field images showing the morphology of primary BM-MSCs and donor-matched iMSCs. **(C)** Flow cytometry analysis of canonical MSC markers (CD45, CD73, CD90, and CD105) in primary BM-MSC and donor-matched iMSC. **(D-E)** Colony-forming unit fibroblast (CFU-F) assays were performed to compare the colony-forming ability of primary MSCs and donor-matched iMSCs. Representative images of CFU-F assays and quantification are shown (n = 3, mean ± SEM; non-significant (ns)).

Figure S2. Full-length image of Western Blot assay.

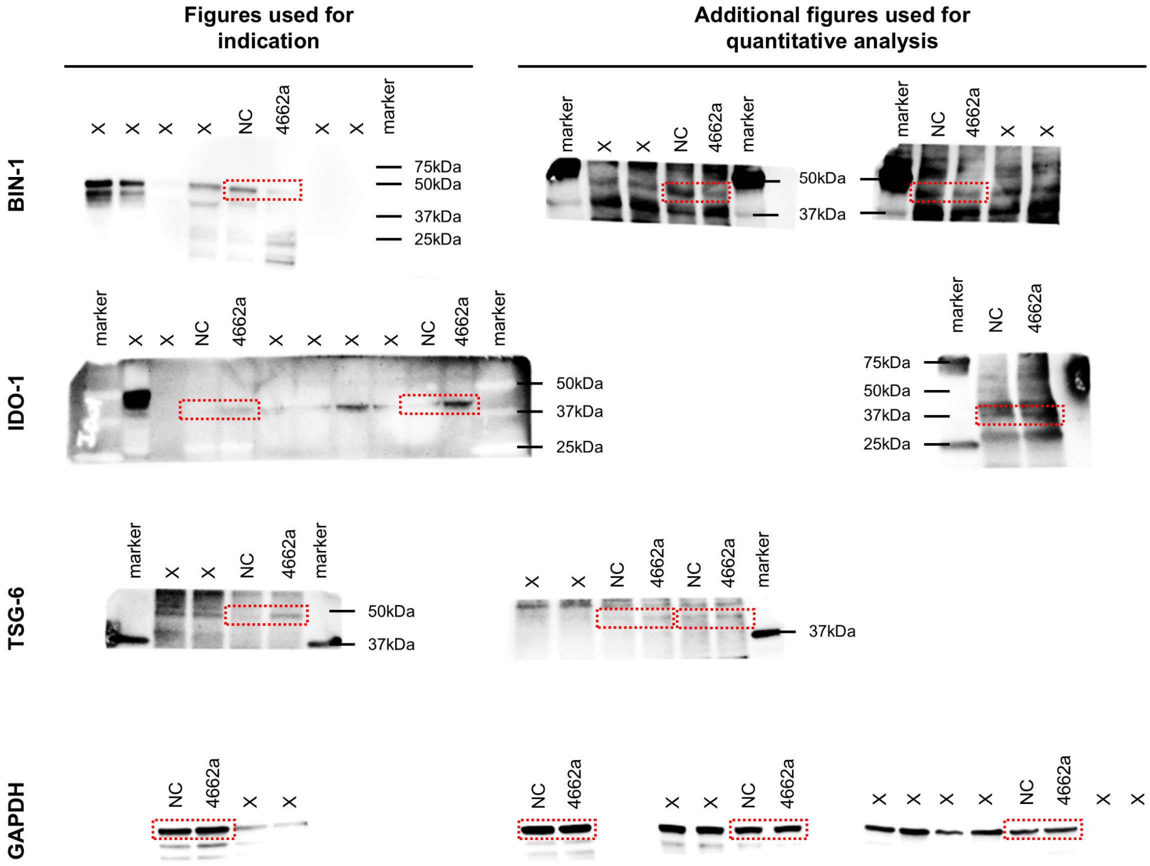

Figure S3. KEGG pathway analysis for effects of miR-4662a on GVHD activity.

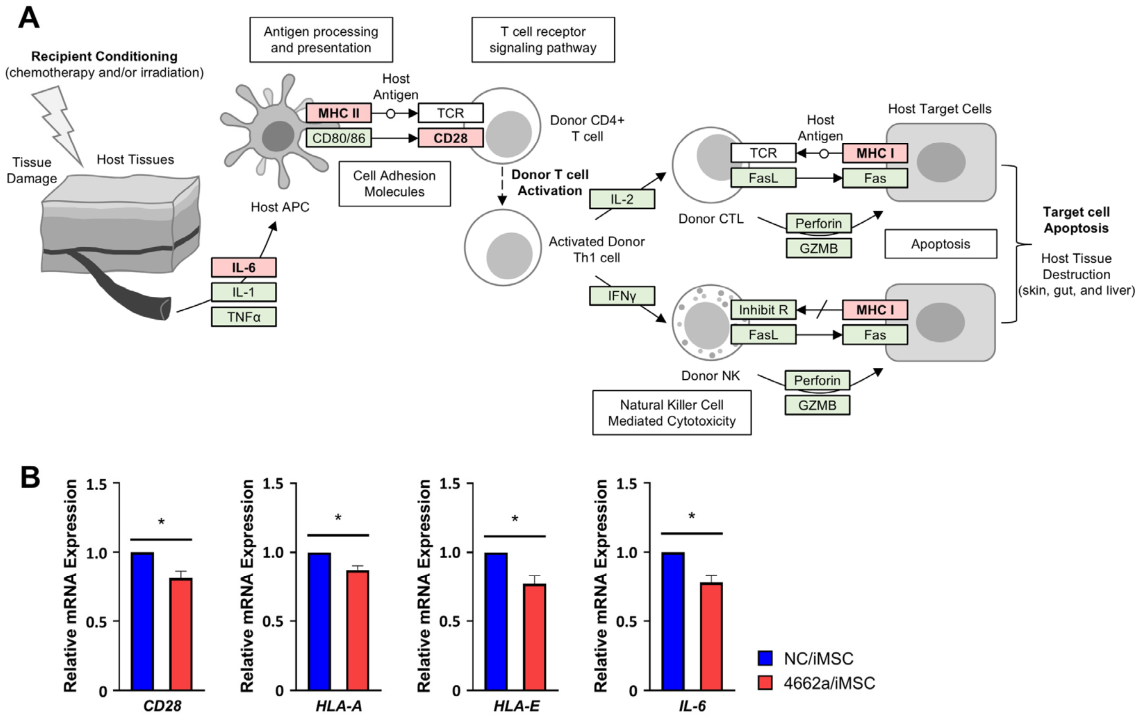

(A) KEGG pathway analysis for graft-versus-host disease (hsa05332). Genes targeted by miR-4662a are marked in red, while human-specific genes are highlighted in green. **(B)** Expression levels of genes predicted to influence GVHD through miR-4662a (*CD28*, *HLA-A*, *HLA-E*, and *IL-6*) were analyzed by RQ-PCR in NC/iMSC or 4662a/iMSCs (n=3 or 4, *; $p < 0.05$). Gene expression levels were normalized to *HPRT* and are shown as fold changes relative to scrambled controls.

Figure S4. miR-4662a regulates key immunomodulatory factors in iMSCs.

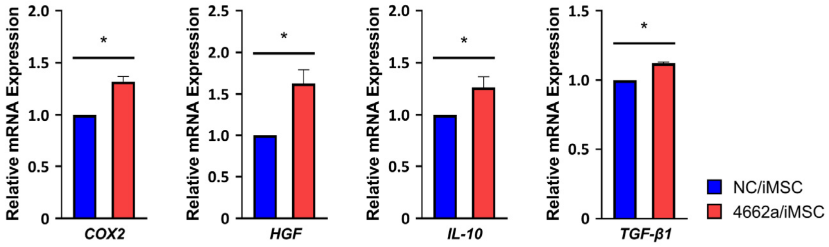

Expression levels of *COX2*, *HGF*, *IL-10*, and *TGF-β1* were assessed using RQ-PCR in iMSCs transfected with miR-4662a (n=4, *; $p < 0.05$). Gene expression levels were normalized to *HPRT* and presented as fold changes relative to scrambled controls.

Figure S5. Immune modulating effects of miR-4662a on T-cells after 10-day culture.

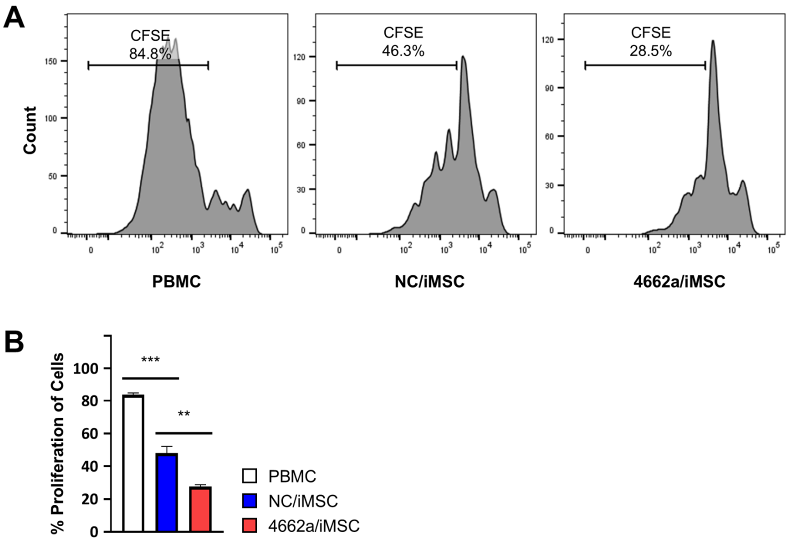

(A-B) Inhibition of T-cell proliferation by 4662a/iMSCs. Peripheral blood mononuclear cells (PBMCs) from normal donors were stained with CFSE and incubated in the absence of iMSCs (PBMC) or co-cultured with NC/iMSC or 4662a/iMSC for 10 days. The decrease of the CFSE intensity by mitotic divisions of T-cells was analyzed by flow cytometry after gating for T-cells (CD3) (CFSE- cells, n=3, **; $p < 0.01$, ***; $p < 0.001$).

Table S1. miRNA sequences used to transfect MSCs for miRNA screening.

miRNA	Sequence
hsa-miR-7-5p inhibitor	5’-AACAACAAAAUCACUAGUCUUCCA-3’
hsa-15b-3p inhibitor	5’-UAGAGCAGCAAAUAAUGAUUCG-3’
hsa-miR-29b-3p inhibitor	5’-AACAVUGAUUUCAAAUGGUGCUA
hsa-miR-29b-1-5p inhibitor	5’-UCUAAACCACCAUAUGAAACCAGC-3’
hsa-miR-128-1-5p inhibitor	5’-UCUCAGACAGUGCUACGGCCCCG-3’
hsa-miR-195-3p inhibitor	5’-GGAGCAGCACAGCCAAUAUUGG-3’
hsa-miR-301a-5p inhibitor	5’-AGUAGUGCAAUAAAGUCAGAGC-3’
hsa-miR-335-3p inhibitor	5’-GGUCAGGAGCAAUAAUGAAAAA-3’
hsa-miR-485-3p inhibitor	5’-AGAGAGGAGAGCCGUGUAUGAC-3’
hsa-miR-503-3p inhibitor	5’-CCUGGCAGCGGAAACAAUACCCC-3’
hsa-miR-573 inhibitor	5’-CUGAUCAGUUACACAUCACUUCAG-3’
hsa-miR-1228-3p inhibitor	5’-GGGGGGCGAGGCAGGUGUGA-3’
hsa-miR-1910-5p inhibitor	5’-AGGCGGCAGGCACAGGACUGG-3’
hsa-miR-3136-5p inhibitor	5’-AAUGACCCUACCUAUUCAGUCAG-3’
hsa-miR-3176 inhibitor	5’-CCGGUAGUCCCAGGCCAGU-3’
hsa-miR-3187-3p inhibitor	5’-CCGCGCAGCCCCAUGGCCAA-3’
hsa-miR-3614-5p inhibitor	5’-GGGCAGCCUUCAGAUCCAAGUGG-3’
hsa-miR-4521 inhibitor	5’-CUGAGCACAGGACUUCCUUAGC-3’
hsa-miR-146b-3p mimic	5’: GCCCUGUGGACUCAGUUCUGGU<br>3’: ACCAGAACUGAGUCCACAGGGC
hsa-miR-146b-5p mimic	5’: UGAGAACUGAAUUCCAUAGGCUG<br>3’: CAGCCUAUGGAAUUCAGUUCUCA
hsa-miR-320c mimic	5’: AAAAGCUGGGUUGAGAGGGU<br>3’: ACCCUCUCAACCCAGCUUUU
hsa-miR-320d mimic	5’: AAAAGCUGGGUUGAGAGGA<br>3’: UCCUCUCAACCCAGCUUUU
hsa-miR-584f-5p mimic	5’: UGCAAAAGUAAUCACAGUUUUU<br>3’: AAAAACUGUGAUUACUUUUGCA
hsa-miR-1246 mimic	5’: AAUGGAUUUUUGGAGCAGG<br>3’: CCUGCUCCAAAAAUCCAUU
hsa-miR-1248 mimic	5’: ACCUUCUUGUAUAAGCACUGUGCUAAA<br>3’: UUUAGCACAGUGCUUAUACAAGAAGGU
hsa-miR-1291 mimic	5’: UGGCCCUGACUGAAGACCAGCAGU<br>3’: ACUGCUGGUCUUCAGUCAGGGCCA
hsa-miR-4448-mimic	5’: GGCUCCUUGGUCUAGGGGUA<br>3’: UACCCCUAGACCAAGGAGCC
hsa-miR-4662a-5p mimic	5’: UUAGCCAAUUGUCCAUCUUUAG<br>3’: CUAAAGAUGGACAAUUGGCUAA

Table S2. qPCR primers used for detecting expression of the gene of interest.

Gene of Interest	Sequence
human *HPRT*	Forward: 5′-CATTATGCTGAGGATTTGGAAAGG-3′<br>Reverse: 5′-CTTGAGCACACAGAGGGCTACA-3′
human *IDO-1*	Forward: 5′-GCCTGATCTCATAGAGTCTGGC-3′<br>Reverse: 5′-TGCATCCCAGAACTAGACGTGC-3′
human *BIN-1*	Forward: 5’-CGTCAACACGTTCCAGAGCATC-3’<br>Reverse: 5’-CTTGACCGTGAAGGTGTTGCTC-3’
human *TSG-6*	Forward: 5′-TCACCTACGCAGAAGCTAAGGC-3′<br>Reverse: 5′-TCCAACTCTGCCCTTAGCCATC-3′
human *EXOSC6*	Forward: 5'-AGGGCTGCCAGCGCCTCTAC-3'<br>Reverse: 5'-CTTGCGTCCGTAGTTGCTCAGG-3'
human *NFKBIZ*	Forward: 5'-CCGATTCGTTGTCTGATGGACC-3'<br>Reverse: 5'-GCACTGCTCTCCTGTTTGGGTT-3'
human *SWAP70*	Forward: 5'-ACTGGAGGAAGCAGCATCTCGT-3'<br>Reverse: 5'-GAGCAACCTGTTCTTCCATCTGC-3'
human *TRAF3IP2*	Forward: 5'-GAGGATAGAATCCGAGGCATTGA-3'<br>Reverse: 5'-GTAAGCCATGCTCATCCTCGTC-3'
human *CD28*	Forward: 5'-GAGAAGAGCAATGGAACCATTATC-3'<br>Reverse: 5'-TAGCAAGCCAGGACTCCACCAA-3'
human *HLA-A*	Forward: 5'-AGATACACCTGCCATGTGCAGC-3'<br>Reverse: 5'-GATCACAGCTCCAAGGAGAACC-3'
human *HLA-E*	Forward: 5'-CGGCTACTACAATCAGAGCGAG-3'<br>Reverse: 5'-AATCCTTGCCGTCGTAGGCGAA-3'
human *IL-6*	Forward: 5’-AGACAGCCACTCACCTCTTCAG-3’<br>Reverse: 5’-TTCTGCCAGTGCCTCTTTGCTG-3’
human *COX-2*	Forward: 5'-CGGTGAAACTCTGGCTAGACAG-3'<br>Reverse: 5'-GCAAACCGTAGATGCTCAGGGA-3'
human *HGF*	Forward: 5'-GAGAGTTGGGTTCTTACTGCACG-3'<br>Reverse: 5'-CTCATCTCCTCTTCCGTGGACA-3'
human *TGF-β1*	Forward: 5'-TACCTGAACCCGTGTTGCTCTC-3'<br>Reverse: 5'-GTTGCTGAGGTATCGCCAGGAA-3'
human *IL-10*	Forward: 5'-TCTCCGAGATGCCTTCAGCAGA-3'<br>Reverse: 5'-TCAGACAAGGCTTGGCAACCCA-3'

Gene symbols

HPRT: hypoxanthine phosphoribosyltransferase 1

IDO-1: indoleamine 2,4-dioxygenase-1

BIN-1: bridging integrator-1

TSG-6: tumor necrosis factor-inducible gene 6

EXOSC6: human exosome component 6

NFKBIZ: NF-kappa-B inhibitor zeta

SWAP70: switch-associated protein 70

TRAF3IP2: adapter protein CIKS

CD28: cluster of differentiation 28

HLA-A: human leukocyte antigen A

HLA-E: human leukocyte antigen E

IL-6: interleukin 6

COX-2: cyclooxygenase-2

HGF: hepatocyte growth factor

TGF-β1: transforming growth factor beta 1

IL-10: interleukin 10

Table S3. Gene Ontology of miR-4662a.

GO Biological Process	Gene number	Expected Value	Over /Under	Fold Enrichment	P-Value	FDR
Detection of stimulus (GO:0051606)	44	122.08	-	0.36	1.17×10^{-18}	1.96×10^{-15}
Polyol metabolic process (GO:0019751)	35	17	+	2.06	1.04×10^{-5}	2.45×10^{-3}
Production of molecular mediator of immune response (GO:0002440)	8	26.59	-	0.3	1.21×10^{-5}	2.82×10^{-3}
Response to leukemia inhibitory factor (GO:1990823)	36	17.72	+	2.03	1.52×10^{-5}	3.38×10^{-3}
Golgi organization (GO:0007030)	47	25.86	+	1.82	2.51×10^{-5}	4.87×10^{-3}
'de novo' IMP biosynthetic process (GO:0006189)	6	1.09	+	5.53	3.49×10^{-5}	6.43×10^{-3}
Endoplasmic reticulum to Golgi vesicle-mediated transport (GO:0006888)	40	22.61	+	1.77	1.58×10^{-4}	2.39×10^{-2}
Immunoglobulin mediated immune response (GO:0016064)	15	33.64	-	0.45	1.64×10^{-4}	2.43×10^{-2}
XMP metabolic process (GO:0097292)	6	1.27	+	4.74	2.06×10^{-4}	2.81×10^{-2}
Maintenance of blood-brain barrier (GO:0035633)	17	7.05	+	2.41	2.00×10^{-4}	2.91×10^{-2}
Epidermal cell differentiation (GO:0009913)	18	37.08	-	0.49	2.39×10^{-4}	3.23×10^{-2}
